# Occupational stressors and its organizational and individual correlates: A nationwide study of Norwegian ambulance personnel

**DOI:** 10.1186/1471-227X-8-16

**Published:** 2008-12-02

**Authors:** Tom Sterud, Erlend Hem, Øivind Ekeberg, Bjørn Lau

**Affiliations:** 1 Department of Behavioural Sciences in Medicine, Institute of Basic Medical Sciences, Faculty of Medicine, University of Oslo, PO Box 1111 Blindern, NO-0317 Oslo, Norway; 2 National Institute of Occupational Health, Oslo, Norway

## Abstract

**Background:**

High levels of stress among ambulance personnel have been attributed to the conditions of ambulance work. However, there is little research to support this notion, and it has been questioned whether ambulance work is inherently stressful. We compared the severity and frequency level of organizational and ambulance-specific stressors, and studied their relationship to organizational conditions and individual differences

**Methods:**

A comprehensive nationwide questionnaire survey of ambulance personnel (n = 1180) in operational duty. The questionnaire included the Job Stress Survey, the Norwegian Ambulance Stress Survey, the Basic Character Inventory, General Self-Efficacy Scale, and questions addressing organizational conditions.

**Results:**

Serious operational tasks and physical demands were identified as the two most severe stressors. Lack of support from co-workers was the most severe and frequent organizational stressor. Higher frequency of stressors was most strongly associated with size of service districts (beta ranging between .18 and .30, *p *< .01) and working overtime (beta ranging from .13 to .27, *p *< .05). Stressor severity was related to lack of support after exposure to critical event (beta ranging from .11 to .24, *p *< .01) and working overtime. Neuroticism (beta ranging from .09 to .17, *p *< .01) and low general self-efficacy (beta ranging from -.12 to -.16, *p *< .001) were equally strongly related to severity of stressors, as were organizational conditions.

**Conclusion:**

Ambulance-specific stressors were reported as both more severe and more frequently occurring stressors than were organizational stressors. Organizational working conditions were more strongly related to frequency of job stressors than were individual differences. In general, the relationship between occupational stressors and individual differences was weak.

## Introduction

Research has indicated that ambulance personnel suffer from symptoms related to traumatic events, and experience more chronic stressors in their work than workers in other health service settings [1]. Thus, it is important to increase our understanding of the type of stressors that may increase the risk of psychological stress symptoms in ambulance personnel.

Much research on health in the ambulance service has been based on the assumption that such work is inherently stressful [2]. Ambulance workers frequently have to take rapid action and provide medical care under life-and-death circumstances in unfamiliar and inconvenient conditions, while being scrutinized by bystanders and relatives [3]. Ambulance personnel also must attend to non-emergency work, such as transporting and providing appropriate care to chronically and terminally ill patients, which imposes different emotional demands and which might be experienced as more emotionally exhausting than more sensational events [4]. Others have claimed that ambulance work may not be inherently stressful, and that it is sources other than ambulance work, such as the 'managerial role', the 'relations with others at work', and the 'home and work relationship' which create pressure for ambulance personnel [5,2]. However, research concerning both administrative-organizational and ambulance-specific stressors is sparse [6].

A potentially important aspect, which has been given little attention, is the distinction between frequency and severity of events. Most studies have considered only the degree of exposure to a stressor [7], without taking into consideration that some situations in ambulance work, such as 'incident with seriously injured children' or 'handling seriously injured persons', may be experienced as very severe stressors that predispose ambulance personnel to post-traumatic stress symptoms. In comparison, administrative-organizational stressors may be experienced as more frequent and chronic stressors. Furthermore, administrative-organizational stressors may not be an expected part of ambulance work and a high frequency level may over time be an important source of frustration and psychological distress in ambulance personnel. Thus, the distinction between frequency and severity of events may be instrumental in evaluating the relative importance of ambulance-specific versus general organizational stressors.

High levels of stressor exposure may be moderated by organizational factors, such as work conditions, and by individual factors, such as personality. Knowledge of such factors provides a basis for preventive efforts. Most studies of ambulance personnel have been limited to a single organization or a single city [8]. Hence, little is known about the ways in which the organizational characteristics of the ambulance services are associated with exposure to, and experience of, stressors. The size of the service population is one potentially important source of stressors that has not been addressed. Ambulance workers may face different challenges in urban and rural areas, such as differences in the closeness of interaction with the client population, distance to the nearest hospital, number and types of incidents, and overall activity level. Furthermore, long working hours or working overtime [9] and shift work [10,11] has been reported to be associated with adverse health consequences, and are postulated to coincide with high job demands [12]. Working overtime increases the time that a worker is exposed to other sources of workplace stress, but may also imply that the duration of effort investment is prolonged, whereas the time left for external recovery is shortened [13]. Thus, working overtime and shift work may sensitize persons to other sources of stress. Social support, on the other hand, is an organizational factor that has been assumed to potentially reduce the level of stress [14]. A peer support system has been formally implemented in some ambulance districts in Norway. To be a peer counsellor, a one-week training course has to be undertaken, and thereafter, a yearly follow-up course. The intention of peer support is to help colleagues with advice in relation to problems in daily work, especially after exposure to serious events.

Some authors have suggested that individual characteristics might explain the high level of distress symptoms among ambulance personnel [15-17]. In general, personality has been postulated to influence stress levels, partly through having an effect on the frequency of exposure to stressors, but more importantly, through modifying the experience of stress severity associated with the stressors [18,19]. Generalized self-efficacy and neuroticism have been reported to be among the best dispositional predictors of job satisfaction and performance [20]. Individuals with high self-efficacy are postulated to deal more effectively with difficulties and persist in the face of failure [21], whereas highly neurotic persons are found to interpret more situations as threatening or damaging [22]. Furthermore, the effect of potentially important sociodemographic variables is not well understood [8]. Being female in a male-dominated working environment such as the ambulance services may be a risk factor for higher levels of job stress among ambulance women. Older employees, on the other hand, are more experienced and may therefore experience potentially traumatic stressors as less severe.

Based on this background information, we conducted a study to address the level of stress (frequency and severity) associated with ambulance-specific and general organizational stressors in a nationwide sample of operational ambulance personnel. We developed the following hypotheses:

1. Ambulance personnel report a generally high frequency and severity level of stressors. Ambulance specific stressors are experienced as most severe, whereas general organizational stressors appear more frequently.

2. Higher levels of stressor frequency are associated with working in more densely populated areas, working overtime and lack of social support. Furthermore, we wanted to explore the possibility that individual characteristics, gender and age are associated with differences in stressor exposure.

3. Higher levels of stressor severity are associated with higher levels of neuroticism and lower levels of self-efficacy. Furthermore, we expected lack of support to be associated with stressor severity, and wanted to explore the possibility that working overtime, and shift work are associated with stressors severity. Lastly, we wanted to explore the possibility that female and younger personnel report higher levels of stressor severity.

## Materials and methods

### Sample

Ambulance services in Norway are organized into 19 main ambulance regions that are responsible for ensuring adequate ambulance services for all communities. Some hospitals have their own integrated ambulance departments, whereas, in several counties, ambulance services are organized independently of the hospital. In several rural locations, ambulance services cover small populations. Here, they consist of one or two ambulances that are usually privately administered. However, private providers have established larger service units in some counties.

Ambulance personnel received no formalized education until 1997. Thereafter, ambulance education consisted of a four-year education course at high-school level (including a two-year apprenticeship). Those who have achieved this qualification can apply for two-year part-time paramedic education at college level. Hence, the educational background of the ambulance personnel in Norway ranges from those with no formal education through to workers with formal ambulance education at high school or college level. Some of the ambulance personnel are also nurses or auxiliary nurses. There were no significant differences in stressor level among respondents with education at high school level or lower compared to respondents with education at high school level (Multivariate Analysis of variance (MANOVA): F = 0.8, hypothesis degrees of freedom = 14, error degrees of freedom = 1153, *p *= 0.64). Education was therefore not applied as an independent variable.

In April 2005, questionnaires were distributed to the chiefs of ambulance services in all 19 ambulance regions in Norway. All chiefs agreed to distribute the questionnaire to all ambulance personnel in the ambulance stations within their regions. This procedure was chosen because no central national register covering all employed ambulance personnel in Norway was available. Respondents were given an identification number in order to enable a follow-up one year later. Two follow-up reminders were distributed through the ambulance chiefs, and the two major worker union organizations encouraged their members to answer the questionnaire in their homepages and their membership journals. In total, 3200 questionnaires were distributed. Based on reports from four of the ambulance chiefs, 64 ambulance personnel were excluded because they were no longer in service. In total, 1286 persons returned questionnaires (41%). Unfortunately, we were not able to get fully updated address lists from the other ambulance chiefs. Thus, the real response rate was probably higher than 41%.

Participants in this study included officers, middle managers and managers who reported ambulance work to be more than 50% of their workload. The term "operational ambulance personnel" is used to describe these respondents. Of the respondents, 1180 were operational ambulance workers (> 50% of their working time). Of these, 76.8% were men and 23.2% women. Age ranged from 18 to 66 years and the mean age was 36.8 (SD 9.3) years. The mean age for men was 37.6 (9.0) years, which was significantly higher than the mean age for women of 33.8 (9.6) (d.f. = 1143, t = 5.96, *p *< 0.001). Multivariate Analysis of variance (MANOVA) was used to compare mean levels on the included outcome variables in those who responded in the main round and those who responded after one and two reminders. No significant differences were found (F = 1.3 hypothesis degrees of freedom = 28, error degrees of freedom = 1980, *p *= 0.14).

After listwise deletion of respondents with missing values on one or more variables, usable responses were obtained from 1,005 (32% of the total sample) operational ambulance personnel, which was the final sample used in the regression analyses. Mean scores on severity and frequency of stressors, after deletion of missing values, did not differ from the estimated mean scores based on all values. Thus, the results are not likely to have been affected by missing values.

### Measures

#### Severity and Frequency of general stressors

General organizational stressors was measured with the Job Stress Survey (JSS) [23]. The instrument consists of 30 items that describe a core set of situations that are encountered in a wide variety of occupations. Each of the 30 stressors is rated on a nine-point perceived severity and frequency rating scale from 0 to 9+, in relation to the last six months. Twenty of the 30 items in the JSS have been reported to constitute the two main dimensions: job pressure and lack of support. However, in different samples, two to three additional factors have been identified [24], and little information is available on the (factorial) validity of the scale [7]. We performed a principal component analysis with varimax rotation to explore the total pool of the 30 items. The 10 items constituting the 'job pressure' dimension were resolved as two factors that gave good conceptual meaning: 'time pressure' (e.g., 'lack of breaks') and 'challenging job tasks' (e.g., 'delegation of increased responsibility'). Of the items considered to constitute the 'lack of support' dimension, 'poorly motivated co-workers', 'co-workers not doing their job' and 'covering work for others' formed a separate factor. Thus, we applied a slightly modified four-factor Spielberger model: 'time pressure' (five items α = .82), 'challenging job tasks' (five items, α .78), 'lack of leader support' (six items, α = .88), and 'lack of co-worker support' (three items, α = .78) (Table 1). A similar factor structure was also supported for the frequency items (not shown).

**Table 1:** Factor loadings in a principal component analysis (Kaiser's varimax rotation) on the 'Job Stress Survey' and 'The Norwegian Ambulance Stress Survey' single items

The Job stress Survey (JSS) Items	Lack of support leaders	Demanding job tasks	Time pressure	Lack of support co-workers
No participating in decisions	**0.84**	*0.20*	*0.03*	*0.04*
Lack recognition for good work	**0.80**	*0.14*	*0.03*	*0.28*
Poor or inadequate supervision	**0.61**	*0.28*	*0.13*	*0.18*
Difficult working with superior	**0.81**	*0.16*	*0.17*	*0.19*
Inadequate support by supervisor	**0.57**	*0.24*	*0.19*	*0.25*
Difficulty getting along with supervisor	**0.79**	*0.01*	*0.19*	*0.22*
Working overtime	*0.16*	**0.56**	*0.05*	*0.12*
Critical on the spot decisions	*0.20*	**0.73**	*0.16*	*0.10*
Assigned increased responsibility	*0.32*	**0.62**	*0.19*	*0.11*
Assignment of new duties	*0.11*	**0.75**	*0.24*	*0.03*
Perform duties not in job description	*0.04*	**0.67**	*0.33*	*0.16*
Frequent interruptions	*0.26*	*0.28*	**0.53**	*0.42*
Frequent change simple to demanding tasks	*0.06*	*0.44*	**0.61**	*0.16*
Excessive paperwork	*0.09*	*0.19*	**0.79**	*0.12*
Meeting deadline	*0.06*	*0.20*	**0.82**	*0.14*
Insufficient personal time	*0.24*	*0.12*	**0.66**	*0.04*
Fellow workers not doing job	*0.26*	*0.21*	*0.01*	**0.77**
Covering work for others	*0.31*	*0.18*	*0.27*	**0.56**
Poorly motivated co-workers	*0.26*	*0.05*	*0.24*	**0.79**
Sums of squared loadings	3.9	2.9	2.9	2.1
% variance	20.4	15.3	15.1	11.0

The Norwegian Ambulance Stress Survey (NASS)		Non-Emergency tasks	Serious operational tasks	Physical demands

Hide feelings towards patients and relatives		**0.83**	*0.13*	*0.16*
Not being able to express own opinion to patients or relatives		**0.82**	*0.08*	*0.19*
Medical responsibility in the vehicle		**0.63**	*0.34*	*0.17*
Negative attitude from relatives about the job you are doing		**0.60**	*0.29*	*0.07*
The cumulative effect of frequently driving chronically ill patients		**0.54**	*0.25*	*0.24*
Take care of seriously injured and dying patients		*0.52*	**0.57**	*0.15*
Uncertainty about what you will meet on the scene of accident		*0.51*	**0.60**	*0.11*
Incident with seriously injured friend or people you know		*0.19*	**0.73**	*0.12*
Incident with seriously injured children		*0.09*	**0.83**	*0.15*
Deal with acting-out and threatening patients		*0.18*	**0.70**	*0.22*
Driving under difficult conditions		*0.34*	**0.62**	*0.18*
Heavy lifting		*0.22*	*0.12*	**0.91**
Carrying out the work under difficult conditions		*0.20*	*0.22*	**0.91**
Working in bent or twisted positions		*0.18*	*0.25*	**0.87**
Sums of squared loadings		3.3	3.2	2.7
% variance		23.3	22.7	19.3

Note. The factor loadings of the items considered to constitute the respective factors are emphasized in bold.

#### Ambulance specific stressors

The Norwegian Ambulance Stress Survey (NASS) was constructed especially for the present study to measure ambulance-specific stressors. In order to establish an adequate list of relevant stressors, two focus group interviews were performed at two ambulance stations with different organizational size and structure. A set of 29 items was described and assessed in the same way as the Job Stress Survey. Nine items tapping co-worker support, and two other items ('high work-load' and 'rumours about changes in the organization') were excluded because they overlapped with the 'lack of co-worker support' and 'challenging job demands' items from the Job Stress Survey. To identify a factor structure of the remaining 18 severity items, we performed a principal component analysis with varimax rotation. This approach resulted in a tree factors solution with eigenvalues (i.e. the variances extracted by the factors) above 1. However, four of the items loaded equally high on two of the factors, and the content of these items was ambiguous with regard to the two factors. Thus, these four items were deleted. The final analysis, based on the 14 remaining items, was resolved as three factors with good conceptual meaning: 'non-emergency tasks' (five items, α = .80), 'serious operational tasks' (six items, α = .85), and 'physical demands' (three items, α = .93). It should be noted that the items "take care of seriously injured and dying patients" and "uncertainty about what you will meet" loaded approximately equally high on two of the factors (i.e. 'serious operational tasks' and 'non-emergency tasks'), however, based on an evaluation of the content of these items, they were included in the serious operational task index (Table 1).

#### Organizational working conditions

The size of the population to be served in the specific ambulance service was measured in five categories: < 5,000; 5,000–20,000; 20,000–50,000; 50,000–100,000; and > 100,000 (reference category). The type of working time was dichotomized: regular working hours (reference category) and shift work. Working overtime was measured with five categories: never (reference category), less than monthly, monthly, every other week, and weekly. Peer support was measured with two questions: 'Has a colleague support system been established at your service place' ('Yes' (reference category); 'Is planned, but not yet implemented', 'No', 'No, but would be nice if there was'), and: 'Have you ever been exposed to a critical event that you would have liked to talk to a colleague about?' ('No' (reference category); 'Not relevant'; 'A few times'; 'Sometimes/often')

#### Individual characteristics

Personality was measured by 27 items from the Basic Character Inventory (BCI), which is based on an original questionnaire constructed by Lazare, Klerman, and Armor [25], and modified by Torgersen [26]. BCI is based on the 'big three' personality dimensions. The BCI – vulnerability scale (α = .74) measures the neuroticism dimension (e.g. I'm very touchy about criticism), the BCI – intensity scale (α = .72) assesses extroversion/introversion (e.g. 'Many people consider me a lively person'), and the BCI – control scale (α = .66) describes the degree of compulsiveness/obsessiveness (e.g. 'Everything I do must be precise and accurate'). Here, the terms neuroticism, extroversion and control were used, respectively. Each dimension is based on nine questions with a dichotomous response (0 = does not apply, 1 = applies), allowing each dimension a range of scores between 0 (low) and 9 (high).

The Generalized Self-Efficacy Scale (GSE) [27] consists of 10 items that assess the strength of an individual's belief in their ability to respond to novel or difficult situations and possible stressors (α = .88). Responses are made on a four-point scale from 1 (not true) to 4 (exactly true). The GSE scale has shown acceptable internal consistency and test-retest reliability [28,29].

Gender was coded with women as a reference category. Age was treated as a continuous variable.

## Results

The product moment correlations among the study variables as well as their means and standard deviations (median and range for categorical variables) and alpha coefficients are provided in a separate table. Alpha coefficients are reported on the diagonal of the correlation matrix for index scores [see Additional file 1]. The mean correlation between frequency and severity indexes was Pearson's r = .32 for the general occupational stressors, and Pearson's r = .16 for the ambulance specific stressors.

A series of paired sampled t-tests were conducted to test the relative ratings of general organizational stressors and ambulance-specific stressors (Table 2). Ambulance specific stressors were reported as significantly more severe than the general organizational stressors. Serious operational demands were reported as the most severe stressor (5.8), and physical demands were the second most severe stressor (5.4). The Non-emergency tasks index, however, was identified as an intermediate stressor (4.4). Among the general work-stress dimensions, lack of support from co-workers (5.4), and leaders (5.1), were identified as significantly more severe stressors than time pressure (4.3) and challenging job tasks (4.4).

**Table 2:** Relative ratings on severity and frequency level of general organizational stressors and ambulance-specific stressors

	Severity items			Frequency items		
	Mean	Significance*	SD	Mean	Significance *	SD
**a) Time pressure**	**4.3**		**1.4**	**2.1**		**2.2**
Frequent interruptions	4.6		1.7	2.0		3.0
Frequent change simple to demanding tasks	4.3		1.7	3.2		3.7
Excessive paperwork	4.1		2.0	1.4		2.5
Meeting deadline	4.2		1.9	1.5		2.7
Insufficient personal time	4.2		2.0	2.2		3.2
**b) Challenging job tasks**	**4.4**		**1.3**	**2.6**	a,c	**1.9**
Working overtime	3.8		1.9	3.9		3.5
Critical on the spot decisions	4.4		1.7	1.5		2.1
Assigned increased responsibility	4.5		1.9	2.2		2.9
Assignment of new duties	4.5		1.8	1.6		2.4
Perform duties no in job description	4.6		1.8	4.0		3.6
**c) Lack of leader-support**	**5.1**	a,b	**1.7**	**2.0**		**2.3**
No participating in decisions	4.8		2.3	1.6		2.5
Lack recognition for good work	5.4		2.1	1.9		2.8
Poor or inadequate supervision	4.9		1.9	2.2		3.1
Difficult working with superior	4.8		2.3	1.5		2.5
Inadequate support by supervisor	5.5		2.3	2.9		3.4
Difficulty getting along with supervisor	5.2		1.9	1.8		2.7
**d) Lack of co-worker support**	**5.4**	a,b,c,e	**1.7**	**3.2**	a,b,c,e,f	**2.9**
Fellow workers not doing job	6.0		1.9	3.7		3.4
Covering work for others	4.7		2.0	2.8		3.2
Poorly motivated co-workers	5.4		2.1	3.0		3.4
**e) Non-emergency tasks index**	**4.4**	a	**1.4**	**2.8**	a,b,c	**2.0**
Hide feelings towards patients and relatives	4.1		1.8	1.8		2.6
Not being able to express own opinion to patients or relatives	4.3		1.8	1.8		2.8
Medical responsibility in the vehicle	4.5		1.8	5.6		4.0
Negative attitude from relatives about the job you are doing	4.9		2.0	1.2		2.1
The cumulative effect of driving chronically ill patients	4.4		1.9	3.7		3.6
**f) Serious Operational tasks index**	**5.8**	a,b,c,d,e	**1.4**	**2.8**	a,b,c	**2.0**
Take care of seriously injured and dying patients	5.2		1.8	4.7		3.5
Uncertainty about what you will meet on the scene of accident	5.2		1.8	3.6		3.2
Incident with seriously injured friend or people you know	6.3		2.0	1.0		1.8
Incident with seriously injured children	6.6		1.8	1.6		2.1
Deal with acting-out and threatening patients	6.0		1.8	1.8		2.3
Driving under difficult conditions	5.3		1.9	4.2		3.6
**g) Physical demands**	**5.4**	a,b,c,e	**1.9**	**5.7**	a,b,c,d,e,f	**3.3**
Heavy lifting	5.0		2.0	6.0		3.6
Carrying out the work under difficult conditions	5.4		2.0	5.3		3.5
Working in bent or twisted positions	5.6		1.9	5.6		3.6

Note. * Each variable (a, b, c, d, e, f, g) was individually tested against all other variables by a series of paired sample t-tests. Level of significance was set at p < .01. A variable with a mean score followed by "a, b" in the significance row indicates that this variable is significant higher than variable a and variable b at p < .01.

The assumption that general stressors appear more frequently than do ambulance specific stressors was not supported. Ambulance specific physical demands were identified as the most frequent stressor (5.7). Moreover, the serious operational tasks index were a significantly more frequent stressor (2.8) than three out of four general work stressors; the exception was lack of co-worker support, which was the second most frequent stressor overall. The three other general stress dimensions (time pressure, challenging job tasks, and lack of leader support) occurred significantly less frequently than were ambulance-specific stressors. Lack of support from leaders was the least frequent stressor.

Multiple linear regressions were used to estimate simultaneous effects of organizational and individual variables on job stress severity (Table 3) and frequency (Table 4). In addition, by removing single blocks of variables from the final model, we estimated the unique contribution of organizational conditions and individual variables (i.e. R^2^, _adjusted, finalmodel _minus R^2^, _adjusted_, _model without organizational and individual variables, respectively_). For each block of variables, the mean unique variance across each stressor dimension, for both frequency and severity, was estimated. Individual variables were equally strongly related to stressor severity as were organizational variables, explaining on average 3.7% and 3.3% of the variance, respectively. Organizational working conditions were more strongly related to frequency of job stressors than were individual differences, explaining on average 10.6% and 1.1% of the variance, respectively. Furthermore, several relationships were explored that are not reported in Tables 4 or 5. There was no statistical support for the assumption that the relationship between the organizational variables and stressors may be moderated by individual characteristics.

**Table 3:** Multiple regressions on the severity dimension (n = 1005)

		Lack of co-worker support		Lack of leader support		Time pressure		Demanding job tasks		Non-emergency tasks index		Serious Operational tasks		Physical demands	
		β	Sig	β	Sig	β	Sig	β	Sig	β	Sig	β	Sig	β	Sig
Gender	Men	0.03	n.s	0.03	n.s	0.12	**	0.07	*	0.05	n.s	-0.07	*	-0.04	n.s
Age		-0.12	**	-0.09	**	0.02	n.s	-0.07	n.s	-0.09	**	0.00	n.s	0.03	n.s
															
Neuroticism		0.17	**	0.14	**	0.06	n.s	0.11	*	0.14	**	0.13	**	0.11	**
Control		0.02	n.s	-0.03	n.s	0.05	n.s	0.02	n.s	0.06	n.s	-0.02	n.s	-0.02	n.s
Introversion		0.04	n.s	0.02	n.s	0.04	n.s	-0.04	n.s	0.02	n.s	-0.05	n.s	0.02	n.s
Self-efficacy		0.08	*	0.07	*	-0.12	**	-0.16	**	-0.16	**	-0.16	**	-0.02	n.s
															
Size of	> 100.000 = ref.														
service	50–100000	0.08	n.s	0.00	n.s	0.03	n.s	0.03	n.s	0.04	n.s	0.00	n.s	-0.04	n.s
population	20–50000	0.08	n.s	-0.06	n.s	0.05	n.s	0.06	n.s	0.10	*	0.04	n.s	-0.05	n.s
	5–20000	0.01	n.s	-0.07	n.s	-0.04	n.s	0.02	n.s	0.07	n.s	0.02	n.s	-0.10	*
	< 5000	-0.09	n.s	-0.13	*	-0.08	n.s	-0.02	n.s	0.00	n.s	0.01	n.s	-0.08	n.s
Work time	Daytime = ref.														
	Shift	-0.03	n.s	-0.04	n.s	-0.09	**	-0.06	ns	-0.06	*	-0.02	n.s	-0.03	n.s
Working	Never = ref.														
overtime	< monthly	0.10	n.s	0.09	n.s	0.06	n.s	0.06	n.s	0.17	*	0.21	**	0.20	**
	monthly	0.15	*	0.09	n.s	0.14	*	0.09	ns	0.21	**	0.27	**	0.20	**
	2 week	0.16	**	0.12	*	0.11	n.s	0.12	*	0.22	**	0.25	**	0.23	**
	weekly	0.17	**	0.08	n.s	0.12	*	0.05	ns	0.17	**	0.20	**	0.24	**
Colleague	Yes = ref.														
support	No	0.02	n.s	0.04	n.s	0.01	n.s	0.06	n.s	-0.01	n.s	0.04	n.s	0.05	n.s
system	No, would be nice	0.02	n.s	0.09	*	-0.03	n.s	0.06	n.s	0.00	n.s	0.09	*	0.09	*
	Planned	0.03	n.s	0.05	n.s	0.02	n.s	-0.01	n.s	0.06	n.s	0.05	n.s	0.04	n.s
'Exposure	No = ref.														
to serious	One or few times	0.11	**	0.12	**	0.11	**	0.11	**	0.11	**	0.16	**	0.06	n.s
Event'	Sometimes	0.14	**	0.16	**	0.20	**	0.14	**	0.15	**	0.24	**	0.15	**
	Not relevant to me	0.01	n.s	0.01	n.s	-0.07	*	-0.02	n.s	-0.05	n.s	-0.02	n.s	-0.07	*

R2		0.09		0.06		0.08		0.08		0.11		0.15		0.06	

*P < 0.05; **P < 0.01; β = standardized beta coefficients; ref. = reference category; Note. The item 'working overtime' was dropped from the index 'challenging job tasks' in the present analysis because 'overtime' was applied as an independent variable leading to an inflated correlation.

**Table 4:** Multiple regressions on the frequency dimension (n = 1005)

		Lack of co-worker support		Lack of leader support		Time pressure		Demanding job tasks		Non-emergency tasks index		Serious Operational tasks		Physical demands	
		β	Sig	β	Sig	β	Sig	β	Sig	β	Sig	β	Sig	β	Sig
Gender	Men	0.02		0.01	n.s	0.03	n.s	0.03	n.s	0.05	n.s	0.09	**	0.05	n.s
Age		-0.16	**	-0.06	*	-0.09	**	-0.19	**	-0.10	**	-0.11	**	-0.09	**
															
Neuroticism		0.11	**	0.15	**	0.05	n.s	0.04	n.s	0.08	*	0.01	n.s	0.03	n.s
Control		0.03	n.s	-0.07	*	0.01	n.s	-0.05	*	-0.04	n.s	-0.05	n.s	-0.05	n.s
Introversion		0.04	n.s	0.05	n.s	-0.02	n.s	0.02	n.s	0.04	n.s	0.02	n.s	0.01	n.s
Self-efficacy		0.14	**	0.13	**	0.09	**	0.09	**	0.12	**	0.13	**	0.09	**
															
Size of	> 100.000 = ref.														
service	50–100000	0.06	ns	-0.01	n.s	-0.06	n.s	-0.06	**	-0.06	n.s	-0.05	n.s	-0.09	*
population	20–50000	0.04	ns	-0.07	n.s	-0.15	**	-0.11	**	-0.12	*	-0.16	**	-0.16	**
	5–20000	-0.10	*	-0.13	**	-0.27	**	-0.18	**	-0.29	**	-0.26	**	-0.26	**
	< 5000	-0.15	**	-0.17	**	-0.30	**	-0.19	**	-0.25	**	-0.25	**	-0.23	**
Work time	Daytime = ref.														
	Shift	0.05		0.02	n.s	0.05	n.s	0.09	**	0.04	n.s	0.08	*	0.07	*
Working	Never = ref.														
overtime	< monthly	0.09		0.01	n.s	0.00	n.s	0.09	**	0.07	n.s	0.11	n.s	0.02	n.s
	monthly	0.16	**	0.03	n.s	0.07	n.s	0.11	**	0.11	n.s	0.17	**	0.10	n.s
	2 week	0.18	**	0.13	*	0.11	*	0.19	**	0.11	*	0.12	*	0.09	n.s
	weekly	0.25	**	0.13	*	0.23	**	0.30	**	0.27	**	0.26	**	0.16	**
Colleague	Yes = ref.														
support	No	0.03	n.s	0.08	*	0.08	*	0.06	n.s	0.08	*	0.06	n.s	0.06	n.s
system	No, would be nice	0.03	n.s	0.11	**	0.03	n.s	0.02	n.s	0.06	n.s	0.04	n.s	0.07	n.s
	Planned	0.06	*	0.05	n.s	0.06	*	0.03	n.s	0.07	*	0.02	n.s	0.06	n.s
'Exposure	No = ref.														
to serious	One or few times	0.14	**	0.08	*	0.10	*	0.10	**	0.12	**	0.11	**	0.08	*
Event'	Sometimes	0.15	**	0.12	**	0.20	**	0.15	**	0.19	**	0.23	**	0.18	**
	Not relevant to me	0.05	n.s	-0.02	n.s	0.03	n.s	0.01	n.s	0.03	n.s	0.07	*	0.02	n.s

R2		0.18		0.09		0.17		0.18		0.15		0.17		0.11	

*P < 0.05; **P < 0.01; β = standardized beta coefficients; ref. = reference category; Note. The item 'working overtime' was dropped from the index 'challenging job tasks' in the present analysis because 'overtime' was applied as an independent variable leading to an inflated correlation.

Table 3 shows that among the organizational variables, working overtime was related to higher levels of severity on all ambulance-specific stressors (β ranging from .17 to .27, *p *< .05) and lack of support from co-workers (β > .15, *p *< .05). 'Exposure to critical event' was consistently related to higher level of stressor severity on all dimensions (β ranging from .11 to .24, *p *< .01). Among the individual variables, neuroticism was related to higher severity scores on all stressor dimensions except for time pressure (β ranging from .09 to .17, *p *< .01), whereas general self-efficacy was related to lower levels of stressor severity on all stressor dimensions (β ranging from -.12 to -.16, p < .001), except physical demands and lack of support from co-workers and leaders. Male personnel reported time pressure (β = .12, *p *< .001) and challenging job tasks (β = .07, *p *< .05) as significantly more severe stressors, but serious operational tasks as less severe stressors (β = .07, *p *< .05), than women. Furthermore, younger personnel reported lack of support from both co-workers and leaders as more severe (β = -.12, *p *< .001 and β = -.09, *p *< .01, respectively),

Table 4 shows that among the organizational variables, working in districts with a population less than 20,000 was associated with lower frequency levels on all stressor dimensions (β ranging between .18 and .30, *p *< .01, except for 'lack of support': β ranging between .10 and .17, p < .05). Furthermore, higher frequencies on all stressor dimensions, were reported among those working overtime weekly (β ranging from .13 to .27, *p *< .05), and among those who had been 'exposed to critical event' sometimes or often (β ranging between .12 and .23, *p *< .01). We did not find any strong relationship between formalized colleague support and lowered stress. However, people who answered 'No, but would be nice if there was' reported significantly higher stressor severity on three out of seven stressors. Among the individual variables, self-efficacy was consistently related to higher frequency of stressor exposure (β ranging from .09 to .14, *p *< .01). Males reported serious operational tasks more frequently (0.09, *p *< .05), and older employees reported overall lower frequencies of stressor exposure (β ranging from -.06 to -.22, *p *< .05).

## Discussion

The assumption that ambulance personnel report a generally high frequency and severity level of stressors was not supported. Most stressors were rated as moderate stressors (close to a scale level of 5), when interpreted within the framework of the Spielberger instrument [23]. Unfortunately, normative data from the general working population in Norway was not available.

In accordance with our hypotheses, ambulance specific stressors were identified as the most severe stressors. Serious operational tasks, and the items 'dealing with seriously injured friends and people you know' and 'dealing with seriously injured children' in particular, were rated as the most severe stressor (a 32% higher mean score than the two general stressors time pressure and challenging job tasks). This is in accordance with what has been pointed out in other studies; some aspects of the job, such as incidents involving children, are especially problematic [4,30,31]. Ambulance specific operational demands may be an expected part of the job and one of the main reasons why these people chose the ambulance occupation in the first hand. However, although ambulance personnel may consider ambulance specific operational demands to be the most meaningful and the most motivating stressors, the high severity stressors may nevertheless be risk factors for post traumatic stress symptoms. Non-emergency tasks, on the other hand, were identified as intermediate stressor on severity level, but were reported as a more frequent stressor than three of the organizational stressors. When looking at item levels, 'medical responsibility in the vehicle' and 'the cumulative effect of frequently driving chronically ill patients' occurred frequently, but with relatively low severity scores (a 32% lower mean severity score than serious operational tasks), which may suggest that ambulance personnel manage to cope reasonably well with these types of stressors.

The assumption that general stressors appear more frequently than do ambulance specific stressors was not supported. Much ambulance work involves heavy lifting and carrying under difficult conditions, and the present data shows that physical demands are the most frequent stressor (a 78% higher mean frequency score than the second most frequent stressor), and the second most severe stressors compared to all other stressors. This concurs with other studies, which have found that ambulance personnel report higher levels of physical strain than employees in other health services [1], and that ambulance personnel self-report more musculoskeletal and physical health problems than the general population [32,33]. Among the general organizational stressors lack of support from co-workers was reported as the second most frequent stressor overall, and lack of support from co-workers and leaders were rated as the most important general stressors evaluated on severity level (a 20% higher mean severity score than time pressure and challenging job tasks). These results concur with other studies that have reported that social aspects of the work environment are associated with higher levels of distress [1,34]. Thus, ambulance specific physical demands and lack of support, especially from co-workers, was rated as relatively high on both severity and frequency level, and may provide a basis for predicting health problems in future studies.

Organizational conditions were moderately related to stressor frequency level, but were significantly more important than were individual characteristics, explaining ten times more of the variance in frequency level. As postulated, working in larger communities was one of the organizational variables most strongly associated with higher frequency of stressor exposure (explaining about 3.3% of the variance in the adjusted model [R^2^,_adjusted_, _finalmodel _≈ 3.3%]). This is likely to reflect the higher number of accidents and incidents in more densely populated urban areas, but with a balanced proportion of ambulance personnel to the number of the service population this does not necessarily have to be the case.

Furthermore, in accordance with our hypotheses, working overtime was significantly related to higher levels of stressor frequency, consistent with what has been reported in other occupational groups [12]. Working overtime was equally strongly related to stressor frequency as were size of the service population and was also significantly related to severity level (R^2^,_adjusted_, _finalmodel _≈ 1.5%), suggesting that working overtime may sensitize personnel to other sources of stress. The assumption that shift-work is associated with higher stressor level was, however, not supported. Shift-work is an integral part of the ambulance services, and most personnel work in shifts. Thus, it cannot be ruled out that the small group who work regular hours is a selected group.

As hypothesized, lack of support after having been 'exposed to a critical event" was significantly related to both higher levels of stressor severity (R^2^,_adjusted_, _finalmodel _≈ 1.5%) and frequency (R^2^,_adjusted_, _finalmodel _≈ 2.3%). On the other hand, we did not find any strong relationship between implementation of colleague support and lower levels of stress. However, people who answered 'No, but would be nice if there was' reported higher severity levels on three out of seven stressors. A possible interpretation is that, although a formalized peer support system may not be essential, it is nevertheless important to have co-workers or leaders to whom one can talk about difficult topics.

Individual characteristics were more strongly related to severity level than to frequency level. In accordance with our hypotheses, ambulance personnel with higher levels of neuroticism (R^2^,_adjusted_, _finalmodel _≈ 1.5%) and low levels of generalized self-efficacy (R^2^,_adjusted_, _finalmodel _≈ 1.2%) appraise work situations as being significantly more severe, consistent with what has been reported in other studies [35,36]. Furthermore, neuroticism was related to both severity and frequency of lack of support from co-workers and leaders. This may indicate that higher levels of daily negative affect lead to interpersonal problems [18], but may also reflect that people with higher levels of neuroticism have a higher need, and therefore do not feel that they receive sufficient social support. On the other hand, personnel with lower levels of self-efficacy appear to experience stressors less often (R^2^,_adjusted_, _finalmodel _≈ 1.1%). Such low frequency ratings may indicate a degree of self-protective behavior; they avoid stressful situations more often than other personnel, but may also reflect that less experience with occupational stressors leads to less confidence in handling these very stressors. Overall, however, there were weak associations between personality and reported stressors frequency and severity. The personality traits control and introversion were not significant in the model. The fact that many of these individual moderators were not significant suggests that work related factors might be stressful in themselves, and may very well be more easily addressed at an organizational level.

In general, there were few differences in reported severity levels associated with gender. However, male personnel reported time pressure and challenging job tasks as significantly more severe stressors (R^2^,_adjusted_, _finalmodel _≈ 1.0%). A similar result was reported in a recent study: women reported lower levels of stress than men, but there was no difference in the frequency with which they encountered it [37]. Further, younger personnel reported lack of support, especially from co-workers as both more frequent and severe stressors (R^2^,_adjusted_, _finalmodel _≈ 2.5% and 1.5%, respectively). Overall, older employees report lower frequency of stressor exposure. A possible interpretation is that older employees have more experience and have learned to cope with potentially stressful incidents more efficiently and may therefore not consider a particular source of stress to be a problem. Younger employees, on the other hand, may be more eager and sensation-seeking [38], and are therefore exposed to stressors more frequently.

### Strengths and limitations

The strengths of this study are that it is one of the largest investigation of ambulance personnel conducted, and it is nationwide. Further, the study applied several validated international instruments, with a large number of respondents making multivariate analyses feasible. The cross-sectional design prevents us from providing direct evidence on the direction of the reported relationships. Organizational variables were measured at individual level and did not take into account that ambulance personnel are nested within groups at station level. The response rate was relatively low, which may question the representativeness of the data. However, there was no difference in the mean levels on the stress indicators between those who returned the questionnaire early, and those who returned it late. As late responders may resemble the non-respondents [39,40], the lack of representativeness may not be a severe problem. Further, because of the problems in the questionnaire distribution, it is likely that the real response rate is higher than the estimated proportion. Lastly, this study focused on relative differences in stress levels within the ambulance services, thus, the level per se was not of critical importance.

## Conclusion

Ambulance-specific stressors were reported as both more severe and more frequently occurring stressors than were organizational stressors. In general, the relationship between occupational stressors, organizational conditions and individual differences was weak. However, this study suggests that working in more densely populated areas was most strongly related to frequency of stressors. Personnel with high levels of neuroticism reported lack of support as both a more severe and frequent stressor. Younger personnel reported lack of support from both co-workers and leaders as more frequent and severe stressors, and reported higher frequency of stressor exposure. Future research may learn more about how ambulance personnel deal with potentially stressful incidents by a greater focus on approaches yielding in depth explorations of ambulance personnel facing stressful conditions over time and across occasions, in the context of their aspirations, beliefs and strategies of coping.

## Competing interests

The authors declare that they have no competing interests.

## Authors' contributions

TS, EH, ØE and BL jointly conceived the idea for the paper. TS performed the statistical analyses. All authors interpreted the data. TS drafted the manuscript. TS will act as guarantor for the paper. All authors approved the final manuscript.

## Pre-publication history

The pre-publication history for this paper can be accessed here:



## Supplementary Material

Additional file 1 **Means, standard deviations, Alpha coefficients+, and Pearson's correlations coefficients between measured variables (N = 1005)**. The product moment correlations among the study variables as well as their  means and standard deviations (median and range for categorical  variables). Alpha coefficients are reported in the diagonal for index  scores. Click here for file
