# Supplementary material for: Occupational stressors and its organizational and individual correlates: A nationwide study of Norwegian ambulance personnel
Source: BMC Emerg Med. 2008 Dec 2;8:16. doi: 10.1186/1471-227X-8-16 (PMC2612695; doi:10.1186/1471-227X-8-16)
Supplement: Additional file 1 — Means, standard deviations, Alpha coefficients+, and Pearson's correlations coefficients between measured variables (N = 1005). The product moment correlations among the study variables as well as their means and standard deviations (median and range for categorical variables). Alpha coefficients are reported in the diagonal for index scores. [file 1471-227X-8-16-S1.doc]

Additional file 1 Means, standard deviations, Alpha coefficients^+^, and Pearson’s correlations coefficients between measured variables (N = 1005)

|  | Mean | SD | Persons's r | |  |  |  |  |  |  |  |  |  |  |  |  |  |  |  |  |  |  |  |  |  |  |
| --- | --- | --- | --- | --- | --- | --- | --- | --- | --- | --- | --- | --- | --- | --- | --- | --- | --- | --- | --- | --- | --- | --- | --- | --- | --- | --- |
|  |  |  | 1 | 2 | 3 | 4 | 5 | 6 | 7 | 8 | 9 | 10 | 11 | 12 | 13 | 14 | 15 | 16 | 17 | 18 | 19 | 20 | 21 | 22 | 23 | 24 |
| 1. Lack of co-worker support_S | 5.4 | 1.6 | .78 |  |  |  |  |  |  |  |  |  |  |  |  |  |  |  |  |  |  |  |  |  |  |  |
| 2. Lack of leader support_S | 5.1 | 1.7 | .51 | .88 |  |  |  |  |  |  |  |  |  |  |  |  |  |  |  |  |  |  |  |  |  |  |
| 3. Time pressure_S | 4.3 | 1.4 | .52 | .43 | .82 |  |  |  |  |  |  |  |  |  |  |  |  |  |  |  |  |  |  |  |  |  |
| 4. Demanding job tasks_S | 4.3 | 1.3 | .41 | .48 | .60 | .78 |  |  |  |  |  |  |  |  |  |  |  |  |  |  |  |  |  |  |  |  |
| 5. Non-emergency tasks index_S | 4.4 | 1.4 | .44 | .39 | .65 | .60 | .80 |  |  |  |  |  |  |  |  |  |  |  |  |  |  |  |  |  |  |  |
| 6. Serious Operational tasks_S | 5.8 | 1.4 | .31 | .27 | .45 | .46 | .65 | .85 |  |  |  |  |  |  |  |  |  |  |  |  |  |  |  |  |  |  |
| 7. Physical demands_S | 5.4 | 1.9 | .24 | .27 | .41 | .38 | .47 | .47 | .93 |  |  |  |  |  |  |  |  |  |  |  |  |  |  |  |  |  |
| 8. Lack of co-worker support_F | 3.2 | 2.9 | .46 | .27 | .10 | .04 | .05 | .03 | .08 | .83 |  |  |  |  |  |  |  |  |  |  |  |  |  |  |  |  |
| 9. Lack of leader support_F | 2.0 | 2.3 | .25 | .56 | .09 | .11 | .06 | .03 | .16 | .52 | .88 |  |  |  |  |  |  |  |  |  |  |  |  |  |  |  |
| 10. Time pressure_F | 2.1 | 2.2 | .24 | .19 | .22 | .06 | .07 | .08 | .14 | .54 | .44 | .75 |  |  |  |  |  |  |  |  |  |  |  |  |  |  |
| 11. Demanding job tasks_F | 2.7 | 1.9 | .24 | .22 | .12 | .06 | .08 | .07 | .10 | .54 | .49 | .61 | .67 |  |  |  |  |  |  |  |  |  |  |  |  |  |
| 12. Non-emergency tasks index_F | 2.9 | 2.0 | .27 | .21 | .11 | .02 | .11 | .09 | .13 | .52 | .41 | .58 | .58 | .66 |  |  |  |  |  |  |  |  |  |  |  |  |
| 13. Serious Operational tasks_F | 2.9 | 2.0 | .19 | .18 | .09 | -.01 | .05 | .06 | .14 | .45 | .40 | .58 | .60 | .71 | .78 |  |  |  |  |  |  |  |  |  |  |  |
| 14. Physical demands_F | 5.8 | 3.2 | .15 | .20 | .11 | .04 | .11 | .13 | .28 | .37 | .31 | .47 | .47 | .57 | .66 | .91 |  |  |  |  |  |  |  |  |  |  |
| 15. Gender (women % / men %) | 23.2% | 76.8% | -.01 | -.01 | .11 | .02 | .00 | -.10 | -.05 | -.01 | -.01 | .03 | .02 | .04 | .10 | .06 | - |  |  |  |  |  |  |  |  |  |
| 16. Age | 36.7 | 9.1 | -.15 | -.13 | .02 | -.05 | -.09 | -.02 | .00 | -.20 | -.12 | -.13 | -.24 | -.15 | -.13 | -.13 | .16 | - |  |  |  |  |  |  |  |  |
| 17. Neuroticism | 2.7 | 2.1 | .16 | .12 | .11 | .17 | .22 | .25 | .14 | .09 | .09 | .05 | .03 | .04 | -.03 | .00 | -.19 | -.07 | .74 |  |  |  |  |  |  |  |
| 18. Control | 3.7 | 2.1 | .03 | -.03 | .03 | .04 | .06 | .04 | .00 | .02 | -.05 | .02 | -.08 | -.04 | -.05 | -.06 | -.11 | .13 | .14 | .66 |  |  |  |  |  |  |
| 19. Extroversion | 5.6 | 2.3 | .02 | .02 | -.02 | -.08 | -.06 | -.11 | -.01 | .07 | .07 | .01 | .06 | .08 | .08 | .05 | -.09 | -.12 | -.27 | -.15 | .72 |  |  |  |  |  |
| 20. Self-efficacy | 3.0 | 0.5 | .04 | .04 | -.13 | -.19 | -.19 | -.22 | -.05 | .14 | .10 | .11 | .12 | .14 | .16 | .12 | .03 | -.05 | -.36 | .03 | .20 | .88 |  |  |  |  |
| 21. Size of service population* | 3 | 1-5 | -.12 | -.10 | -.08 | .02 | -.01 | .07 | -.06 | -.25 | -.12 | -.30 | -.24 | -.25 | -.26 | -.24 | -.07 | .16 | .00 | .04 | -.06 | -.13 | - |  |  |  |
| 22. Working overtime* | 2 | 1-4 | .15 | .08 | .13 | .05 | .11 | .10 | .15 | .24 | .16 | .28 | .29 | .24 | .22 | .19 | .01 | -.01 | .05 | .01 | .00 | .06 | -.15 | - |  |  |
| 23. Shift work* | 2 | 1-2 | .03 | .00 | -.03 | -.04 | -.03 | -.03 | .00 | .14 | .06 | .14 | .18 | .11 | .15 | .14 | .07 | -.02 | -.02 | -.03 | .03 | .00 | -.25 | .13 | - |  |
| 24. Exposure to serious event* | 3 | 1-4 | .14 | .14 | .12 | .10 | .11 | .17 | .13 | .16 | .13 | .18 | .15 | .17 | .21 | .16 | -.03 | .03 | .10 | .05 | .03 | .02 | -.01 | .15 | .04 | - |
| 25. Colleague support system* | 3 | 1-4 | .00 | .03 | -.02 | .09 | .03 | .11 | .04 | -.05 | .05 | -.07 | -.06 | -.05 | -.06 | -.04 | -.05 | -.04 | .01 | .03 | -.04 | -.06 | .39 | -.10 | -.12 | .01 |

_Note. All tests two-tailed_; *Person’s r > .07* significant at p < .05 and *Person’s r > .09* significant at p < .01; S = Severity level and F = Frequency level; Categorical variables are marked with an asterisk (*), these variables are described by their median and range. +Alpha coefficients for index scores are reported in the diagonal.
